# Acceptability and feasibility of the school-engaged social and behavior change communication approach on malaria prevention in Ethiopia: implications for engagement, empowerment, and retention (EER) of education sectors in malaria elimination efforts

**DOI:** 10.1186/s12889-021-11995-z

**Published:** 2021-10-21

**Authors:** Fira Abamecha, Gachana Midaksa, Morankar Sudhakar, Lakew Abebe, Yohannes Kebede, Abebe Mamo, Guda Alemayehu, Zewdie Birhanu

**Affiliations:** 1 Department of Health, Behavior, and Society, Faculty of Public Health, Institute of Health, Jimma University, P.O.Box: 378, Jimma, Ethiopia; 2 Department of Public Health, Mizan-Tepi University, College of Medicine and Health Sciences, Mizan-Aman, Ethiopia; 3 USAID/Ethiopia office, Addis Ababa, Ethiopia

**Keywords:** SBCC, School, Malaria, Feasibility, Acceptability, Stakeholders, Ethiopia

## Abstract

**Background:**

Evidence on process outcomes such as acceptability, and feasibility of behavior change communication interventions are important in program evaluation to understand how, and why such a program works. However; documented evidence on the issue is not available as far as the social and behavior change communication (SBCC) on malaria is concerned. Enrolling the frontline providers this study measured the acceptability and feasibility of the school-engaged SBCC strategy on malaria prevention in malaria-endemic settings of Ethiopia.

**Methods:**

A school-engaged SBCC strategy involving various communication and capacity-building interventions aimed to advance malaria preventive practices in primary schools in Jimma were implemented from 2017 to 2019. A cross-sectional study was conducted with 205 key stakeholders at the end of the intervention. Both acceptability and feasibility were measured using standardized tools. Data were collected using a structured questionnaire and filled by the study participants. The SPSS version 26 was used to analyze the data. Multivariate general linear modeling was performed to identify the predictors of acceptability and feasibility of the program. P-value < 5% was considered to decide statistical significance.

**Results:**

The result showed the mean scores (M, range = R) of acceptability and feasibility of the program were (M = 25.63, R = 6 to 30) and (M = 19.35, R = 5 to 25) respectively. The multivariate linear modeling showed acceptability was affected by self-efficacy; (β = 0.438, P < 0.001), community support; (β = 0.417, P < 0.001), school climate; (β = − 0.16; P = 0.003), perceived malaria threat; (β = 0.40, P < 0.001) and knowledge; (β = 0.229, P = 0.013). Similarly, feasibility was influenced by self-efficacy; (β = 0.352, P < 0.001), community support; (β = 0.591, P < 0.001), school climate; (β = − 0.099, P-value < 0.030) and perceived malaria threat; (β = 0.172, P = 0.002).

**Conclusion:**

With a considerably high level of acceptability, the school-engaged SBCC strategy to enhance malaria preventive practices seems feasible. The SBCC strategy targeting personal factors such as malaria threat perceptions, knowledge and skills on the program, and contextual factors that include school social climate and community support would be fruitful to facilitate the implementation of the program. The result implicates the benefit of intensifying such a strategy to engage, empower, and retain the education sectors in malaria elimination efforts and beyond.

**Supplementary Information:**

The online version contains supplementary material available at 10.1186/s12889-021-11995-z.

## Background

Historical records indicated that malaria is the oldest deadly disease of man-kind that is transmitted by mosquito species called anopheles mosquito [1]. With an estimated, 229 million in 2019 malaria remained the most important global public health issue [2]. The global malaria cases remained unchanged over the last 4 years causing the loss of 409,000 lives in 2019, 411,000 lives in 2018, 451, 000 in 2017, and 435, 000 in 2016 [2]. More than 70% of Ethiopia is a malaria-risk area and about 52% of the population of the country is at risk for malaria [3].

Malaria is one of the diseases of man-king that received more global attention for elimination within the coming few decades [4–7]. However, the inadequate emphasis on community participation and stakeholders’ engagement while extreme reliance on the costly malaria elimination approaches seems paradoxical [8]. The disrupted health services connected to the Covid-19 pandemic and the emerging malaria resurgence, the upcoming years are expected to demonstrate stagnation in malaria reduction. Therefore, promoting collective actions through the involvement of the community and local institutions such as schools is important to accelerate the global and national malaria elimination efforts [6]. One of the cost-effective strategies to engage and empower the community towards malaria control actions is the use of SBCC [5, 6].

The SBCC strategy requires the use of diverse approaches including mass media and interpersonal communications, community participation, and multisectoral engagement aimed at influencing community norms and organizational systems in support of individual behaviors change for health improvement [9]. The SBCC has been effectively used to advancing community participation in the previous malaria prevention and control programs [6, 10]. Key to behavioral change, literature showed that the SBCC was found effective in promoting the key malaria preventive behaviors in the target community [11, 12]. An active engagement, empowerment and retention of the frontline stakeholders, community, and local institutions such as schools in health interventions are major components of the SBCC [9, 13]. Specifically, evidence indicated that the school-based interventions have multiplier effects on community’s health outcomes in which the students and teachers have greater credibility to influencing the families, neighbors, and friends towards healthy practices, though empirical evidence to this regard is extremely limited as far as malaria is concerned [14].

Nevertheless; evidence on ways through which the schools and education sectors can effectively be engaged in the malaria preventive efforts is limited as far SBCC approach is concerned. This calls for process evaluation researches that explicitly link the implementation processes to the effects of the intervention [13, 15–17]. Key to process evaluation; constructs of implementation outcomes such as acceptability and feasibility are indicators that mediate between the intervention and its ultimate effects [18]. Acceptability and feasibility are the two key measures of the process outcomes important to a wide range of implementation researches [19]. They are assessed or measured in several ways including the stakeholders’, providers’, and program target perspectives or perceptions towards the program under consideration [18, 19].

Evidence on the feasibility and acceptability of a program is useful to understand the mechanism by which the program produces or led to the effects. While studies on the acceptability, and feasibility of various health promotion strategies are increasingly numerous in scientific pieces of literature, those targeting students with the school-based SBCC approach on malaria preventive actions remain fragmented [20]. Moreover, there is no long-standing experiences implementation of the SBCC strategy in Ethiopia partly due to its recent emergence [3, 21]. Thus, to our knowledge, no study has been conducted to evaluate the acceptability and feasibility of the SBCC strategy on malaria prevention in malaria-endemic settings of Ethiopia. This study is one of the several studies [22–24]; aimed at measuring the acceptability and feasibility of the school-engaged malaria SBCC in Ethiopia to broadly understand the success of the program.

Focusing on the perspectives and experiences of frontline providers/stakeholders; the current study sought to answer; 1) what is the level of acceptability and feasibility of the school-engaged malaria SBCC in primary schools in rural Ethiopia? 2) What are the socio-demographic, cognitive, schools organizational and social supports factors affecting the acceptability and feasibility of the target program? Drawing on the extensive field experiences of the frontline personnel; the current study provided a valuable input that has implications for guiding interventions on how to embed the malaria SBCC strategy into the existing health care, education, and school system to ultimately enhance effectiveness, ownership, and maintenance.

## Methods and materials

### Study setting

The current study was part of the previous studies conducted in five districts of Jimma where malaria transmission rate was relatively high. Data was collected between May 10 to 30/2020 [22, 23]. The aim of the study was to evaluate the acceptability and feasibility of the school-engaged malaria SBCC intervention targeted to advancing the community’s and schools’ on practices malaria prevention [23]. Importantly, the project was designed considering critical local and national contexts or situations such as literacy, media, and health policy system statuses relevant to the SBCC practices in Ethiopia.

### Literacy and media situation

Education is one of the major socio-economic factors that determine the individuals’ healthy behaviors and attitudes. Ethiopia is one of the developing nations to have little or no education. The demographic and health survey (DHS 2016) showed that about half (48%) of females and nearly one in four (28%) of males have never attended school in Ethiopia. About four in every ten females (39%) had only some primary education. Only 4% of females and 5% of males have attended but not completed secondary education or higher education [25].

Interestingly, access to media and exposure to health information has an important role to promote health-related knowledge, attitude, and practices; though this was quite low in Ethiopia. There is the complex relationship between literacy and access to media which has important implications for designing and implementing the SBCC program. According to DHS 2016, nearly 68% of women and 53% of men aged 15–49 were not exposed to any mass media. Nearly three in four (74%) women and 62% of men have no access to radio, television, or newspapers weekly [25]. Furthermore, relatively radio was the most accessible kind of media in the community of Ethiopia. Specific to Jimma (the study area of the current program), an earlier study (that was conducted in 2013) showed that only 53.5% of the households had a radio [26].

### Health care system

Historically, the primary health care system in Ethiopia was characterized by the heavy emphasis on top-down approaches which failed to consider cultural diversity and true community participation [27]. However, the recent health system in Ethiopia was organized into *three-tier* systems classifying the health care delivery structures as primary, secondary, and tertiary levels. The aim was to effectively reach residents with effective and basic preventive and curative services under the umbrella of *health for all* [28]. The health system was connected to the community or housholds through the health posts. The health posts are the smallest formal structure which is staffed by the health extension workers (HEWs) [29, 30]. Connected with the ultimate goal of the SBCC strategy that mainly drew on community engagement to influence norms and reinforce specific behaviors; it was believed that the an understanding of the existing health care system and community networks can help to design and promote malaria preventive communication using the school-engaged SBCC strategy.

### Intervention

The development of the intervention was informed by evidence from formative qualitative research, which was conducted to explore the local malaria situation and map the relevant partners and stakeholders. The finding of the formative qualitative research was used as the baseline for the development of the school-engaged malaria SBCC packages (“participant enrichment”) and to design the quantitative research instruments (“instrument validity”) that was used in the end-line evaluation survey. The aim of the intervention was to engage, empower and retain the target institutions and the key personnel in the community on collaborative design and implementation of malaria prevention and control efforts. The program employed multiple strategies aligned in hierarchies or iterative processes to reach the local residents and primary schools with malaria preventive messages and recommended actions. Several step-by-step participatory activities such as training, strengthening the existing social networks, providing necessary materials, equipment, and communication guides were implemented [23]. Finally, the roles and responsibilities of various stakeholders, (Table/Supplementary File 1)”, and organizational linkages, (Figure/Supplementary File 2)” were built in order to enhance collaborative works, accountability, continuous provision of monitoring, feedback and supports.

### Study design and participants

A cross-sectional study was conducted with 205 individuals or key services providers to collect retrospective data at the end of the intervention. All activities of the project such as consolidation, sustainment, exit strategy, and handing-over were accomplished before the actual data collection. The participants were frontline personnel recruited from schools and public health organizations that include all frontline staffs who were collaborated to implement the program under consideration [23].

### Data collection tools and methods

The data were collected using a structured questionnaire, (Questionnaire/Supplementary File 3)” that was adapted from previous evidence [18, 19, 31, 32]. The acceptability and feasibility were the two outcome variables of this study that were captured using standardized scales designed based on recommendation [33]. The constructs measured the perceptions and experiences of the frontline personnel on the program benefits, operational and technical challenges, the settings and expected intervention’s effects. Accordingly, acceptability was defined as the beliefs about the extent to which the school-engaged malaria SBCC was satisfactory in fulfilling the local needs and their expectations [31, 32]. It was measured using six items with declarative statements (e.g. this program would benefit the local needs on malaria issues) formatted on a 5-point Likert scale such that 1 = strongly disagree, to 5 = strongly agree [19]. The internal consistency of the items yields an acceptable range of Cronbach’s alpha (α = 0.84). Items were summed up in which the high composite score was interpreted as the high acceptability of the program.

Similarly, the feasibility of the program was operationalized as the extent to which the school-engaged malaria SBCC can be successfully carried out within a given agency or settings [19]. It taps into the practical component of the intervention and helps to understand how easily it can be implemented under given resources, skills and settings it’s being delivered [33]. This was measured using five items (α = 0.73) with declarative statements *(e.g this program seems easy to implement in the school setting)* formatted on a 5-point Likert scale such that *1 = strongly disagree, to 5 = strongly agree.* A high composite score indicated higher feasibility.

Moreover, this study also measured the perceptions of the participants on critical factors such as the schools and community settings to explore the organizational and social supports using measures adapted from different kinds of literatures [34, 35]. Finally, measures of the psychosocial factors knowledge on malaria preventive measures, self-efficacy towards the program, and perceived malaria threats were also addressed in this study [36–39]. Except for knowledge; that was measured using open-ended questions, all the remaining constructs of this study were measured using items formatted on 5-point Likert scales [23].

### Validity and reliability of the constructs

The content and face validity of the psychometrically measured constructs were ensured by adapting the tool from previous related studies. The English version questionnaire was translated to the local language to facilitate understanding. The internal consistency was ensured by using multiple items to measure each construct and this was validated by performing item analysis for internal consistency using the Cronbach’s alpha (α) with an acceptable level greater than 0.6. Data were collected by trained personnel and the process was closely supervised.

### Data analysis

Data were analyzed using the SPSS version 26 software for analysis. Frequency, proportions, and means with standard deviation were computed as part of descriptive analysis. The relationship between the selected predictors of acceptability and feasibility was analyzed using multivariate general linear modeling (GLM). The GLM was recommended method of statistical modeling of multiple continuous dependent variables that are conceptually correlated or covary [40]. This study measured two correlated dependent variables/constructs with continuous values (i.e. acceptability and feasibility). Therefore; the multivariate GLM was appropriately chosen to account for the possible covariance that exists between these psychological variables [40]. Weighted regression coefficients (beta, β) were interpreted to indicate the association between the predictors and dependent variables. A *P*-value of less than 5% was considered to decide statistical significance.

## Results

### Socio-demographic factors

With two hundred and five; (205) participation rates, the study yields a high response rate, 98.10%. The teacher was the major profession of the participants; 137 (66.83%) while male participants and age between 25 and 29 years account for the majority with 130 (63.41%) and 109 (53.17%) respectively. The Oromo ethnic group represents the majority of the participants’ ethnic category, 190 (92.68%). The protestant and Muslim religious categories account for higher with 78 (38.05%) and 49 (23.90) respectively. The majority of the participants were married; 173 (84.39%) and have a monthly salary of 4000-4999birr; 95 (46.34%). Participants who reported diploma as the highest level of education attained and experience of 6–10 years account majority with 150 (73.17%) and 126 (61.46%) respectively. More than half of the participants; 119 (58.05%) received some sort of health-related training. Table 1.

**Table 1:** Relationships of the socio-demographic characteristics and mean scores of acceptability and feasibility of the school-engaged malaria SBCC, Jimma, Ethiopia 2019, (*N* = 205)

Characteristics		Mean score (SD)	
	Frequency (%)	Feasibility	Acceptability
Districts			
Gera	40 (19.80)	11.08(2.18)	25.88 (2.85)
Shebe Sombo	41 (20.29)	12.72(1.58)	26.17(3.57)
Nono Benja	38 (18.81)	12.18(1.78)	25.37(3.76)
Botor Tollay	40 (19.80)	11.84(2.25)	24.68 (4.20)
Limmu Kossa	43 (21.29)	12.49(1.76)	26.00(4.16)
*P*-value		0.002	0.379
Job category			
Teachers^b^	137 (66.83)	12.08(2.03)	25.85(3.73)
Health workers	68 (33.17)	11.96(1.97)	25.09(3.72)
*P*-value		0.677	0.172
Age			
< 24 years	11 (5.37)	13.18()1.47	27.18(2.96)
25–29 years	109 (53.17)	12.13(1.89)	25.52(3.69)
30–34 years	72 (35.12)	11.69(2.24)	25.40(4.13)
> 35 years	13 (6.34)	12.15(1.57)	25.92(1.89)
*P*-value		0.110	0.513
Sex			
Male	130 (63.41)	11.87(2.09)	25.71()3.91
Female	75 (36.59)	12.33(1.81)	25.39(3.44)
*P*-value		0.111	0.545
Ethnicity			
Oromo	190 (92.68)	12.03(2.04)	25.54(3.84)
Others^a^	15 (7.32)	12.13(2.00)	26.27(1.94)
*P*-value		0.850	0.471
Religion			
Orthodox	49 (23.90)	11.86(1.94)	25.06(4.17)
Muslim	78 (38.05)	12.09(2.07)	25.51(3.44)
Protestant	78 (38.05)	12.10(2.00)	26.01(3.74)
*P*-value		0.768	0.367
Marital status			
Single	32 (15.61)	12.50(1.72)	26.03(3.29)
Married	173 (84.39)	11.95(2.05)	25.51(3.82)
*P*-value		0.157	0.474
Level of education			
Diploma	150 (73.17)	12.23(1.94)	25.79(3.34)
BSc degree	55 (26.83)	11.51(2.10)	25.05(4.64)
*P*-value		0.022	0.211
Experiences			
< 5 years	21 (10.23)	12.19(2.11)	25.76(3.85)
6–10 years	126 (61.46)	12.21(1.87)	25.83(3.45)
> 11 years	58 (28.29)	11.60(2.22)	25.02(4.27)
*P*-value		0.148	0.381
Monthly salary			
< 2999birr	34 (16.59)	12.53(2.15)	25.56(4.40)
3000-3999birr	37 (18.05)	12.32(1.33)	26.30(3.00)
4000-4999birr	95 (46.34)	11.92(2.15)	25.52(3.62)
> 5000birr	39 (19.02)	11.64(1.98)	25.15(4.08)
*P*-value		0.197	0.597
Received training on a health-related issue			
Yes	119 (58.05)	12.16(1.96)	25.59(3.56)
No	86 (41.95)	11.87(2.07)	25.60(3.99)
*P*-value		0.312	0.975

Others^a^ = Amhara, Kafa, Guraghe, *SD* standard deviations, Teachers^b^ = this include all school personnel such as schools’ principals and malaria focal teachers.

### Descriptive parameters and Pearson’s correlation coefficients (r)

The descriptive statistical result showed that higher mean scores (M, R = range) of the measures of acceptability and feasibility of the SBCC strategy are above and over the expected average values. The mean acceptability and feasibility of the program were (M = 25.63, range = 6 to 30) and (M = 19.35, range = 5 to 25) respectively. Similarly, both measures of acceptability and feasibility were significantly and positively correlated with each other, *(r = 0.41, P < 0.001*). Moreover; except for knowledge, all other measures of psychosocial factors were significantly correlated with at least one of the measures of feasibility and acceptability. Finally, school climate construct was correlated only to acceptability (r = − 0.26, *P* = 0.001) while the community support significantly correlated to both acceptability and feasibility (r = − 0.16, *P* = 0.05). Table 2.

**Table 2:** Pearson’s correlation coefficients (r) for the measures of acceptability and feasibility of the school-engaged malaria SBCC approach among stakeholders in Jimma, 2020, (*N* = 205)

S.N	Constructs	1	2	3	4	5	6	7
1	Perceived malaria threat	1	.03	.18^a^	−.11	−.01	.14^a^	−.02
2	Community support		1	.08	−.15^a^	.01	.16^a^	.16^a^
3	School climate			1	−.03	−.10	−.13	−.23^b^
4	Knowledge				1	.04	.08	.06
5	Self-efficacy					1	.29^b^	.15^a^
6	Feasibility						1	.41^b^
7	Acceptability							1

Key: ^a^Correlation is significant at the 0.05 level (2-tailed), ^b^.Correlation is significant at the 0.01 level (2-tailed)

### Correlates of feasibility and acceptability

Multivariate GLM was conducted with predictors that were significantly associated with the either acceptability or feasibility (dependent variables) in the bivariate analysis to identify the potential predictors. The level of education was the only sociodemographic factor that was significantly associated with at least one of the dependent variables. However; this relationship becomes insignificant in the multivariate modeling. The other predictors such as perceived malaria threat, self-efficacy, school climate, and community support were all associated with at least one of the dependent variables on multivariate analysis.

Accordingly; acceptability was affected by self-efficacy; (β = 0.438, *P*-value< 0.001), perceived community supports; (β = 0.417, *P*-value< 0.001), school climate; (β = − 0.16; *P*-value = 0.003), perceived malaria threat; (β =0.40, *P*-value< 0.001) and knowledge on EMAs; (β = 0.229, *P*-value< 0.013). This implies the higher belief or confidence; knowledge and community support on SBCC combined with the high perception of disease threat would improve acceptability while this relationship is reduced by the higher positive beliefs about the school climate (individuals’ perception about the existing relationship, leadership, communication in the schools). That’s, the stakeholders who hold strong beliefs about the positive influence of the school climate tend to be more skeptical to confess the program as appealing/acceptable.

Similarly; factors such as self-efficacy; (β =0.352, *P-value < 0.001*), community support; (β =0.591, *P-value < 0.001*) and perceived malaria threat; (β =0.172, *P-value = 0.002*) were positively associated with feasibility of the intervention. The school climate; (β = − 0.099, *P-value* < *0.030*) was also negatively associated with the feasibility of the intervention. The feasibility (practicality) of the program would be enhanced by individuals’ factors such as malaria threat perceptions, confidence, and community support in implementing the program. Finally, the positive perception about the existing school climate would decrease the feasibility of the intervention. Table 3.

**Table 3:** General linear modeling parameters for the predictors of accepatability and feasibility of the school-engaged malaria SBCC approach among stakeholders, Jimma (*N* = 205)

Dependent variables	Predictors	Coeffs. (β)	*SE*	*P-value*
Feasibility				
	Age in years	0.099	0.056	0.081
	Level of education (diploma)^a^	0.110	0.486	0.105
	Experience in years	−0.026	0.082	0.755
	Knowledge	0.010	0.005	0.062
	Self-efficacy	0.210	0.039	0.000
	Perceived malaria threat	0.125	0.053	0.020
	Community support	0.232	0.103	0.025
	School climate	−0.099	0.045	0.030
Acceptability				
	Age in years	0.782	0.892	0.382
	Level of education (diploma)^a^	0.074	0.642	0.909
	Experience in years	−0.564	1.305	0.666
	Knowledge	0.229	0.091	0.013
	Self-efficacy	0.438	0.041	0.001
	Perceived malaria threat	0.400	0.095	0.001
	Community support	0.417	0.138	0.001
	School climate	−0.160	0.129	0.003

Key: ^**a**^Level of education: has two categories; Diploma = 1 and BSc degree = 0, *SE* = standard error.

## Discussion

This study measured the acceptability and feasibility of the school-engaged malaria SBCC aimed to advance malaria prevention and control practices in malaria-endemic settings of Ethiopia. To our knowledge, the study is the first in Ethiopia that examined the acceptability and feasibility of the SBCC on malaria prevention enrolling the frontline stakeholders. Accordingly, the result showed that with a considerably adequate level of delivery and acceptability, this communication program on malaria seems feasible. Moreover, multiple individuals and contextual factors affecting the acceptability and feasibility of the program were identified in this study.

Specifically, the high level of acceptability and feasibility of the SBCC strategy indicated in this study implied that the strategy was more appealing and practically suitable to addressing the malaria situation in the study area. Although; the cut-off points for sound interpretation are not yet available; literature suggested that the relatively high scores indicate greater acceptability and feasibility [19]. The improved acceptance in the current study might be connected to the higher community (recipients) acceptance as reported in one of the previous studies aimed to evaluate the same program (i.e. school-engaged SBCC) [24]. A study examining the acceptability and feasibility of an intervention targeted to promoting physical activity behaviors in primary school reported a similar result [41]. A study conducted in Ghana to evaluate the acceptability and feasibility of the school-based intervention directed to strengthening the reproductive health information and services indicated support and approval from the teachers and health workers [42]. However; it’s true that discrepancy between acceptability and feasibility may often exist in real-world settings. For instance, a study showed that a program that was perceived appropriate was not feasible due to the disparity in resources and contextual requirements of the settings [33]. The findings of this study imply the school’s potential to reach the local communities with malaria preventive messages and actions [14].

Moreover, a wide array of factors related to individual beliefs, perception about the community support, and school climate, affecting the acceptability and feasibility of the intervention, were identified in this study. Specifically, the knowledge in essential malaria control measures and confidence in the ability to run the intervention positively influenced both acceptability and feasibility. Our finding is comparable with the result of a previous study that indicated staff who feel more confident in their ability to implement what was expected in the program had better acceptance and implemented more [43]. Another study showed that skills gaps in community involvement in national school health policy affected the implementation feasibility among the school’s principals [44]. It was also indicated that the capabilities such as procedural knowledge, skills, and motivation affected the shared decision-making interventions on contraceptive care among clinical and administrative staff [45]. This finding implies the importance of emphasizing or building the knowledge and skills on the health communication program and processes to practically engage, empower and retain (EER) the key partners and stakeholders.

The individual factors or personal belief of malaria threat was positively associated with both feasibility and acceptability of the intervention. In support of this result; the cognitive-behavioral theories indicated that the high threat perception (perception of risk plus severity) are potential factors that drive or motivate people towards implementing risk alleviating actions [46–48]. People are more motivated to approve and participate in disease preventive actions when they perceive that they are vulnerable to severe disease. The possible reason for the observed relationships in this study might be due to the fact that the current study was conducted in malaria-endemic settings and this might have affected the threat perception of malaria. However; it was reported that malaria incidence is showing a decreasing trend in Ethiopia that could reduce the public’s perception of the disease [8, 24].

Moreover, evidence of implementation researches suggested that interventions that are grounded in positive organizational climates such as inspiring leadership, open communication, participatory decision making, and positive social supports have greater acceptance and better feasibility [43, 49]. Previous implementation studies of various health and behavior change programs in the schools showed the positive effects of the school climate on the intervention processes and its effectiveness [50–52]. However, the current study showed paradoxical findings in that the school climate was negatively associated with the acceptability and feasibility of the intervention. Participants who felt positive towards the school settings and support tended to be more skeptical about the program and its feasibility. A consistent finding was reported from a previous study in which teachers’ positive perceptions about the existing decision-making climates in the school negatively affected the intervention delivery and acceptance [52].

The complex interplay of multiple factors such as the nature of the program (strategy), nature of settings, staff turnover, providers’ or receipts’ attitude, interests, and expectations may be responsible for the observed paradoxical finding in the current study. The imbalance between expectations and actual fulfillment of the school setting appropriateness for better decision-making process or the failure to aligning their expectations with the goal of the program was associated with reduced perceptions of the program relevance and acceptability [43]. Moreover, the mediating effects of attitudes and beliefs about the program might also be attributed to these observed variations. For example, it was shown the effect of school climate on school-wide physical activities (PA) intervention delivery was mediated by teachers’ attitude toward PA and the beliefs about their responsibility to undertake the programs [52, 53]. The reason behind this result may be due to some misperceptions or misunderstandings in the schools or health systems such as *“who is responsible to address the health issues”, “do schools are really qualified for health care?”* [23]. However, this complex relationship must be further explored using better research designs such as longitudinal studies.

he other most important factor positively affecting the acceptability and feasibility of this study was community support. The perceived community support represents the individual perception about the influence of aspects of community contexts including the existing culture, community connections/networks, and social supports on the implementation and continuity of the target program [54]. The providers exist within wider social contexts that shape, support, or constrain their actions as they tend to interact within organized settings such as community networks, faith organizations, and social service agencies that influence their collective norms and sets of routines of health care practices. It was shown that addressing conditions emanating from the community, promoting community acceptance and ownership enhances the uptake and adoption of a program [43]. However; this is not always the case as enhanced community coalition and recruitment efforts were negatively related to its implementation outcomes in the previous study [55]. The positive effect of the perceived community supports in this study can be supported by the result of similar study (i.e. evaluating the effectiveness of the school-based malaria SBCC) which reported high level of community acceptance and adoption [24].

Finally, none of the socio-demographic characteristics of the study participants were associated with either feasibility or acceptability. A comparable result was reported from the previous review that indicated the demographic factors generally have small or no effects on the acceptability of environmental policies [56]. The finding of this study implies that the variations in the level of the acceptability and feasibility of the program are mainly explained by factors related to the cognitive and behavioral skills dimension (e.g. knowledge, self-efficacy) and not by the socio-demographic characteristics. The possible reason may be the study participants were all qualified professionals and self-sufficient in which their preferences, behaviors, or actions are not shaped by the variations in the socio-demographic characteristics.

### Implications

The current study demonstrated that the school-engaged malaria SBCC strategy was adequately received or accepted by the key personnel for its benefits in addressing the malaria situations in the area. Further, it was believed that the intervention was technically and operationally feasible. Therefore, this implies that we could intensify the SBCC strategies to accelerate the global and national malaria elimination goals [3, 21, 57], by taking scale up measures such as enrolling several villages, schools (i.e. primary, secondary, and higher institutions), involving more stakeholders, and partners from the public and private hospitals, non-governmental organizations, and faith-based organizations. The lesson learned from this study may have practical implications for advancing the existing school health practices in general and malaria preventive communication in particular. Adoption of the SBCC strategy in schools could also improve the existing school health education approach that mainly lacks real community and stakeholders engagement [58].

The current study provided a valuable input that has methodological implications for guiding interventions on how to embed the malaria SBCC strategy into the existing health care, health education and school system to ultimately enhance effectiveness, ownership and maintenance. The study suggested how process evaluation of the malaria SBCC strategy would be done to generate evidence that could contribute to the advancement in the implementation research practices. Finally, the study provides an insight on how the program process measures such as feasibility and acceptability can be measured; as potential indicators (the main challenges in the process evaluation) to understand the overall success of the SBCC strategies [59, 60].

### Strength and limitations

The result of the current study was based on the data collected from the self-report of practices or behaviors and not based on an observational data of implementation process. The self-report data may be subjected to the social desirability bias that may overestimate the treatment integrity as compared with observational methods to collect objective data [61]. However; many studies have shown a correspondence between the self-report data and the observational data on depicting the dynamics or nature of the program process [62–64]. It was also recommended that the self-report methods (e.g. perceptions and experiences) about the intervention is more useful to capture the real quality of implementation that couldn’t be explored through the conventional observational data [43].

The finding of the current study might be affected by the possible instability of the psychological states (because of the use of the psychometrical constructed measures) over time and under different situations. Finally, the lack of empirical evidence on process evaluation of the SBCC strategy on malaria was another important limitation of the current study. Thus, the result of the current study was interpreted based on available evidence (e.g. evidence on school-based health behavior change programs).

## Conclusion

With considerably effective delivery and a high level of acceptability, the school-engaged malaria SBCC strategy seems feasible. The result suggested that the strategy was appealing and practically relevant to enhance the malaria preventive practices both in primary schools and villages. The SBCC strategy that targets personal factors such as malaria threat perceptions, knowledge on malaria, and personal skills on the program, and contextual factors that include school climate, school system, and community support would be fruitful to facilitate the implementation and uptake of the program. The result implicates the need for intensifying such a strategy to engage, empower, and retain (EER) the schools in malaria elimination efforts and beyond. To better understand how the improvement in the level of acceptability and feasibility would influence the ultimate effects of the intervention on malaria preventive actions, further longitudinal research involving RCT with a larger sample should be conducted.

## Supplementary Information

**Additional file 1.**

**Additional file 2.**

**Additional file 3.**

## Data Availability

The datasets used and analyzed during the current study are available from the corresponding author on reasonable request.

## Abbreviations

SBCC: Social and Behavior Change Communication
ITN: Insecticide Treated Nets
IRS: Insecticide Residual Spray
WHO: World Health Organization
EER: Engagement, Empowerment and Retention
CSA: Central Statistical Agency
API: Annual parasite incidence rate
DHS: Demographic and Health Survey
HEW: Health Extension Workers
PHC: Primary Health Care
HLM: Health Learning Materials
EMA: Essential Malaria Actions
GLM: General Linear Modeling
