# Supplementary material for: Acceptability and feasibility of the school-engaged social and behavior change communication approach on malaria prevention in Ethiopia: implications for engagement, empowerment, and retention (EER) of education sectors in malaria elimination efforts
Source: BMC Public Health. 2021 Oct 21;21:1909. doi: 10.1186/s12889-021-11995-z (PMC8529361; doi:10.1186/s12889-021-11995-z)
Supplement: Supplementary file 1 — Additional file 1. [file 12889_2021_11995_MOESM1_ESM.docx]

**Annex 1: Roles and responsibilities**

Summary of roles and responsibility of different actors and stakeholders for the performance of the school based social and behavior change communication (SBCC) intervention on malaria prevention and control in primary schools in Jimma, Ethiopia, 2019.

| **Actors in the project** | **Descriptions of roles in the project** |
| --- | --- |
| Jimma University (the program’s hosting organization) | - Played a catalyst role - Strengthening capacity of schools and local medias through training, discussion, dialogue, supportive supervisions and follow up - Design, develop and provide the IEC/BCC materials - Design and develop malaria communication guides/manuals and materials - Develop monitoring and evaluation plan - Ensure integration of the program into local health system - Regularly monitor the program - Participate in monthly review meeting and other discussions - Conduct surveys and disseminate results at national and international level. - Also produce scientific reports and journal articles. |
| Field officers | - Are recruited health staffs to facilitate the SBCC activities in the communities and schools - Participate in coordination, supervision and monitoring of the program - Participate in training programs - Mobilize schools and religious leaders - Provide communications tools/resources - Own the program - Collects project reports as part of HEP activities - Participate in review meetings and provide feedback |
| Teachers | - Teach students about malaria regularly - Engage, plan, implement and supervise students at peer education activities - Organize and report the peer education and schools malaria communication activities - Demonstrate 4 do-able malaria actions - Teach all teachers about malaria regularly - Facilitate/coordinate malaria education activities within school and in the community - Encourage students to teach their families, neighbors - Mobilize students - Participate in M&E process |
| Students | - Teach/advice their families and neighbors to adopt the 4 do-able actions and eight essential malaria actions. - Advice their friends and peers how to prevent malaria - Participate in malaria prevention activities within school and outside schools such campaign - Participate in community mobilization for prevention and control of malaria |
| Health extension workers | - Closely work with schools and contribute in building schools capacities and establish strong linkage with schools. - Provide technical support, supervise, coordinate and monitor the program - Participate in and lead health education planning by religious leaders and schools - Supplies communication tools for schools and religious leaders - Document/collects, compile and submit reports on project activities as per requirements of HEP reporting system - Participate in monthly meeting at district level - Participate in M&E process |
| District health offices | - Participate in coordination, supervision and monitoring of the program - Participate in training programs - Mobilize schools and religious leaders - Provide communications tools/resources - Own the program - Collects project reports as part of HEP activities - Participate in review meetings and provide feedback - Work closely and coordinate malaria communication activities with district education offices |
| District education offices | - Mobilize students and teachers - Participate in monthly meetings - Support and encourage teachers and students towards working malaria prevention - Participate in malaria prevention campaign - Participate in M&E process - Work closely and coordinate malaria communication activities with district health offices |
| Zonal health department | - Own the program through HEP - Participate in coordination, supervision and monitoring of the program - Attend meetings, sensitization workshops - Collects reports and provide feedback as part of HEP - Supply some communication tools - Scale up best practices and lessons |
| USAID-Ethiopia | - Financially support the program - Oversee the project implementation and financial management according to USAID’s rules and regulations - Collects reports and provide feedback on project implementations - Participate in semi-annual and annual review meetings |
