# Supplementary figures and images for: Acceptability and feasibility of the school-engaged social and behavior change communication approach on malaria prevention in Ethiopia: implications for engagement, empowerment, and retention (EER) of education sectors in malaria elimination efforts
Source: BMC Public Health. 2021 Oct 21;21:1909. doi: 10.1186/s12889-021-11995-z (PMC8529361; doi:10.1186/s12889-021-11995-z)

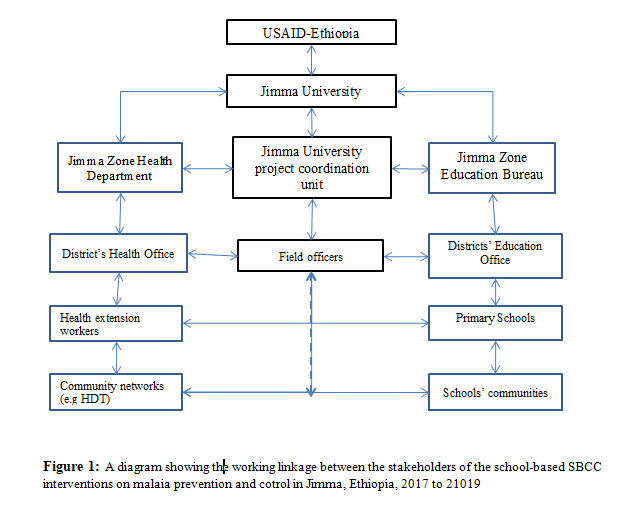

Supplement: Supplementary file 2 — Additional file 2. [file 12889_2021_11995_MOESM2_ESM.docx]
