# Supplementary material for: Acceptability and feasibility of the school-engaged social and behavior change communication approach on malaria prevention in Ethiopia: implications for engagement, empowerment, and retention (EER) of education sectors in malaria elimination efforts
Source: BMC Public Health. 2021 Oct 21;21:1909. doi: 10.1186/s12889-021-11995-z (PMC8529361; doi:10.1186/s12889-021-11995-z)
Supplement: Supplementary file 3 — Additional file 3. [file 12889_2021_11995_MOESM3_ESM.docx]

**Annex 2: Questionnaire**

This questionnaire form has two parts. The first part covers the socio-demographic information and the second part addresses your perceptions and opinion about the implementation fidelity and implementation outcomes of the school-based social and behavior change communication in Jimma. We want you carefully to read each item and give your responses accordingly. Please feel free to ask anything that you are not clear with. Thanks!

**Part I: Socio-demographic characteristics of the participants**

| **s.n** | **Variables** | **Categories/indicators** |  |
| --- | --- | --- | --- |
|  | District | ______________ |  |
|  | Village | ______________ |  |
|  | Altitude (in meter) | ______________ |  |
|  | Name of school/health office | _____________ |  |
|  | Phone number | ________________ |  |
|  | Job category | 1. Teacher 2. Health worker 3. Other, specify |  |
|  | Roles (in school or health care) | _______________ |  |
|  | Profession/department | ____________ |  |
|  | Level of education | 1. Diploma 2. Degree, BSc 3. MSc/MPH 4. Other, specify _____ |  |
|  | Year of recruitment/experience | ____________ |  |
|  | Sex | 1. male 2. female |  |
|  | Age incomplete year | ___________ |  |
|  | Marital status | 1. Single 2. Married 3. Separated/divorced |  |
|  | Religion | 1. Orthodox 2. Muslim 3. Protestant 4. Other, Specify_____ |  |
|  | Ethnicity | 1. Oromo 2. Amhara 3. Others, specify____ |  |
|  | Monthly salary (in birr) | ___________ |  |
|  | Did you receive any health-related training (other than the current program)? | 1. Yes 2. No |  |
|  | Did you receive malaria-related training (other than that of the current program) | 1. yes 2. no |  |
|  | School-level demographics and parameters |  |  |
| 19 | Year of establishment/school age | __________years |  |
| 20 | School size/area (square meter), | ______________Sq. meter |  |
|  | Estimated annual budget | ___________Birr |  |
| 21 | Distance from the main road, | _________KM |  |
| 22 | Presence of sports/football field | 1. Yes 2. No |  |
| 23 | Have functional mass media equipment | 1. tape 2. radio 3. TV 4. Loudspeaker 5. Others |  |
| 24 | Basic facilities | 1. electricity 2. water supply 3. toilets (of any kind) |  |
| 25 | Total No. sections/rooms in school | Mention: __________ |  |
| 26 | Total No. teachers in the school | Mentions____________ |  |
| 27 | Teachers-students ration | Mention:____________ |  |
| 28 | Number of male and female teachers | Male _______and female _____ |  |
| 29 | Degree specification | 1. diploma level___ 2. BSc/BA degree level ____ 3. MSc/MA level______ |  |
| 30 | School directors demography | 1. Sex:_____________ 2. Level of education _______ 3. Experiences ______years |  |
| 31 | Number of students (male versus female) | ____ (M______& F_______) |  |
| 32 | List school level clubs for students | 1. __________ 2. ____________ 3. _____________ 4. ___________ 5. ____________ 6. ____________ |  |
| 33 | Do you have mini-media (education-entertainment) program in your school? | 1. Yes 2. No |  |
| 34 | Do you have ICT centers with internet available in the schools? | 1. Yes 2. No |  |

**PART II:** questionnaire regarding knowledge on essential malaria actions (EMAs), malaria risk perceptions and perceptions/experiences about the intervention delivery and implementation outcomes of the school-based SBCC approach on malaria prevention and control in Jimma, Ethiopia: stakeholders’ perspectives

| D-1 | Knowledge items | Answers (leave this part empty during data collection) | | | | | |
| --- | --- | --- | --- | --- | --- | --- | --- |
|  | Would you please mention the eight essential malaria actions (EMAs) which have been implementing in this community over the last two years?  **NOTE:** The answers will be left empty or blank space will be used to record responses as the participant's mention | 1. All family members sleep under insecticide-treated nets (ITN), every night. 2. Giving priority to pregnant women and children under five to sleep under ITNs, every night. 3. Whenever a family member has a fever, take them to the nearest health facility, immediately. 4. Take a full dose of the anti-malaria drugs prescribed to you by health personnel, including health extension workers (HEWs). 5. Avoid interrupting or sharing the anti-malaria drugs which are prescribed by health personnel 6. Cooperating with sprayers during indoor residual spraying (IRS) period. 7. Avoid re-plastering home for six months after the house has been sprayed. 8. Wash your ITNs with „regular‟ soap and hang or lay to dry in the shade. | | | | | |
| **D-2** | **Perceived susceptibility to malaria:** Now, I ask your beliefs about the likelihood of your exposure to malaria cases in this area. Responses are marked with a Likert scale of 1 to 5; where (1=strongly disagree and 5=strongly agree) that you would rate your opinion about the likelihood of your vulnerability to malaria. [check ‘√” in front of each statement under specified number from 1 to 5] | | **1** | **2** | **3** | **4** | **5** |
| 2 | Malaria appears a common disease (highly prevalent) in this area | |  |  |  |  |  |
| 3 | Malaria appears disappeared or reduced in our village | |  |  |  |  |  |
| 4 | I remember a time when my families have affected by malaria | |  |  |  |  |  |
| 5 | I feel that the chances are high that I can get malaria | |  |  |  |  |  |
| 6 | During the rainy season, I worry every day that I may get malaria* | |  |  |  |  |  |
| 7 | People only get malaria when there are lots of mosquitoes | |  |  |  |  |  |
| 8 | People in this community only get malaria during the rainy season | |  |  |  |  |  |
| 9 | Every year, someone in this community gets malaria infection* | |  |  |  |  |  |
| 9 | A pregnant woman is at risk of malaria than any other person | |  |  |  |  |  |
| **D-3** | **Perceived severity of malaria:** This part seeks to elicit your beliefs about the severity of the bad consequences of malaria such as severe illness, work impairments, economic losses, and death in the worst scenario. Responses are marked with a Likert scale of 1 to 5; where (1=strongly disagree and 5=strongly agree) that you would rate your opinion about how much you feel malaria illness leads to bad outcomes. [check ‘√” in front of each statement under specified number from 1 to 5] | | **1** | **2** | **3** | **4** | **5** |
| 10 | Infections with malaria can potentially lead to death | |  |  |  |  |  |
| 11 | Pain resulting from malaria infection is intolerable to withstand | |  |  |  |  |  |
| 12 | Someone who gets malaria is expected to completely recover in a few days* | |  |  |  |  |  |
| 13 | Malaria infection could seriously harm a pregnant woman and her fetus | |  |  |  |  |  |
| 14 | The malaria illness may reduce my work achievement | |  |  |  |  |  |
| **D-4** | **Competence: self-efficacy**: this explores your confidence or perceived self-efficacy on malaria issues and the SBCC interventions (community mobilization and behavior change skills). Below listed are statements supposed to describe your opinions about how much you rate your skills and knowledge? Would you please indicate the extent to which you agree or disagree based on a 1 to 5 Likert scale; where (1=strongly disagree and 5=strongly agree) to each statement? | | **1** | **2** | **3** | **4** | **5** |
| 15 | I am confident about my abilities for completing the tasks assigned to me | |  |  |  |  |  |
| 16 | I am able to utilize available resources to accomplish tasks | |  |  |  |  |  |
| 17 | I take personal initiatives in carrying out my work. | |  |  |  |  |  |
| 18 | I am well equipped with the skills to develop curricula for the students. | |  |  |  |  |  |
| 19 | I can utilize guidelines available to teach students or the public regarding malaria? | |  |  |  |  |  |
| 20 | I can identify a person with fever/malaria | |  |  |  |  |  |
| 21 | I can handle and care (wash, repair) the ITNs for appropriate use | |  |  |  |  |  |
| 22 | I can dissiminate malaria messages to my school or villages | |  |  |  |  |  |
| 23 | I am confident in my interpersonal skills (on how to work in a team) | |  |  |  |  |  |
| 24 | I can negotiate with school directors to work on malaria action | |  |  |  |  |  |
| **D-5** | **School/organizational climate:** this will elicit your perceptions about the extent to which the organizational systems and social supports within the school or health settings are responsive to the SBCC program. These include perceptions towards school systems, rules, decision making process, supports, and social relations in the schools community. | | **1** | **2** | **3** | **4** | **5** |
| 25 | The relationships with my bosses are good | |  |  |  |  |  |
| 26 | My bosses encourage me when I have problems so that I can solve them | |  |  |  |  |  |
| 27 | My suggestions about the work is listening | |  |  |  |  |  |
| 28 | Opportunities for training are offered | |  |  |  |  |  |
| 29 | If I need help because of a heavy workload, I am given the necessary means | |  |  |  |  |  |
| 30 | The goal of my work are clearly defined | |  |  |  |  |  |
| 31 | The bosses are willing to listen to their employees | |  |  |  |  |  |
| 32 | Socially, my work has the prestige it deserve | |  |  |  |  |  |
| 33 | In my job, innovate contributions are appreciated | |  |  |  |  |  |
| 34 | When I do something well, my siniors congratulate me | |  |  |  |  |  |
| 35 | My work is adequately defined | |  |  |  |  |  |
| 36 | Deadlines are adequately met | |  |  |  |  |  |
| 37 | My bosses watch me closely | |  |  |  |  |  |
| 38 | My work is inadequately supervised | |  |  |  |  |  |
| 39 | Everything is decided from above | |  |  |  |  |  |
| **D-6** | **Percieved community support :** this taps the perceptions about the existing community such as *sense of belongings* (connections), *community participation* (active participation in community activities) and *community organizations* (perceived social support of in the community) that could provide them with opportunities for members of the community to participate in malaria preventive actions. beleow there are nine (9) items with responses rated on a 5-point scale from (1) *strongly disagree* to (5) *strongly agree* to to measure this components. Please indicate the extent to which you agree or disagree to each statement. *How much do you rate the degree to which individuals in your community?* | | **1** | **2** | **3** | **4** | **5** |
| 40 | Identify themselves with their community | |  |  |  |  |  |
| 41 | Individuals’ opinions are valued in the community | |  |  |  |  |  |
| 42 | Feel like their community is their own | |  |  |  |  |  |
| 43 | Do collaborate in organizations and associations in their community | |  |  |  |  |  |
| 44 | Do motivated to take part in social activities in their community | |  |  |  |  |  |
| 45 | Do respond to calls for support in their community | |  |  |  |  |  |
| 46 | Could find people that would help them feel better, | |  |  |  |  |  |
| 47 | Would find someone to listen to me when I feel down, | |  |  |  |  |  |
| 48 | Would they relax and easily forget their problems. | |  |  |  |  |  |
| **D-7** | **Acceptability:** The acceptability of implementation measures (AIM) is defined as the perception of stakeholders that the program (school-based SBCC in this case) practices are satisfactory in fulfilling local needs and expectations. Indicate your level of agreement or disagreement with each statement regarding the program based on a 1 to 5 Likert scale; where (1=strongly disagree and 5=strongly agree). | |  |  |  |  |  |
| 50 | The program satisfactorily meets your/local community needs. | |  |  |  |  |  |
| 51 | I liked the school-based SBCC approach designed to prevent malaria | |  |  |  |  |  |
| 52 | I can give my approval to the school-based SBCC targeted to prevent malaria | |  |  |  |  |  |
| 53 | This school-based SBCC approach meets my/local needs | |  |  |  |  |  |
| 54 | This school-based SBCC approach is satisfactory for the target issue | |  |  |  |  |  |
| 55 | This school-based SBCC approach is appealing | |  |  |  |  |  |
| 56 | I guarantee that the school-based SBCC intervention will do. | |  |  |  |  |  |
| 57 | I believe the program fully belongs to our schools and community | |  |  |  |  |  |
| 58 | I would recommend/ I welcome if such a program is to be implemented in other schools | |  |  |  |  |  |
| **D-8** | **Appropriateness**: The question on intervention’s appropriateness measure (IAM) is designed to explore your perceptions about the fitness, relevance, or compatibility of the school-based SBCC practice on malaria prevention to schools and community settings. Indicate your level of agreement or disagreement with each statement regarding the program based on a 1 to 5 Likert scale; where (1=strongly disagree and 5=strongly agree). | |  |  |  |  |  |
| 59 | This school-based SBCC practice seems fitting to this setting. | |  |  |  |  |  |
| 60 | This school-based SBCC practice seems suitable through school settings. | |  |  |  |  |  |
| 61 | This school-based SBCC practice seems applicable to the existing malaria problems. | |  |  |  |  |  |
| 62 | This school-based SBCC practice has a good match with school systems. | |  |  |  |  |  |
| 63 | This school-based SBCC practice seems well-aligned to the school system. | |  |  |  |  |  |
| 64 | I found participating schools (staffs) in school SBCC approach was appropriate | |  |  |  |  |  |
| **D-9** | **Feasibility**: the feasibility of implementation measures (FIM) explores your opinion/perceptions towards the extent to which the school-based SBCC interventions can be successfully carried out within a given agency (resources, skills) and settings (contexts/system). Indicate your level of agreement or disagreement with each statement regarding the program based on a 1 to 5 Likert scale; where (1=strongly disagree and 5=strongly agree). | |  |  |  |  |  |
| 65 | This school-based SBCC intervention seems practical in this setting | |  |  |  |  |  |
| 66 | This school-based SBCC intervention seems easy to use/implement in this community | |  |  |  |  |  |
| 67 | This school-based SBCC intervention seams realistic (has observable changes) | |  |  |  |  |  |
| 68 | These school-based SBCC intervention components seem implementable | |  |  |  |  |  |
| 69 | This school-based SBCC intervention seems possible (to achieve goals) | |  |  |  |  |  |
| 70 | This school-based SBCC intervention seems challenging/confusing* | |  |  |  |  |  |
